# Technostress in Nursing Education: A Scoping Review

**DOI:** 10.3390/nursrep15070248

**Published:** 2025-07-08

**Authors:** Catarina Lobão, Adriana Coelho, Vitor Parola, Hugo Neves, Joana Pereira Sousa, Rui Gonçalves

**Affiliations:** Health Sciences Research Unit: Nursing, Nursing School of Coimbra, 3045-043 Coimbra, Portugal; catarinalobao@esenfc.pt (C.L.); adriananevescoelho@esenfc.pt (A.C.); vitorparola@esenfc.pt (V.P.); hugoneves@esenfc.pt (H.N.); joanasousa@esenfc.pt (J.P.S.)

**Keywords:** fear of missing out, technostress, nursing education, higher education

## Abstract

Technological advancement has radically transformed higher education, requiring faculty members to continually adapt to new tools and teaching methods. In this context, the phenomenon of fear of missing out (FoMO) has gained relevance, often manifesting through a range of negative emotional states, including technostress—stress associated with the use of technology. **Objectives**: This study aimed to map the available scientific evidence on technostress among nursing faculty in higher education, through a scoping review conducted according to the methodology proposed by the Joanna Briggs Institute (JBI). **Methods**: The literature search was performed across eight databases, including Medline (via PubMed), CINAHL Complete, Scopus, and the Teacher Reference Center. **Results**: Of the 266 studies identified, only 3 met the inclusion criteria. **Conclusions**: Findings reveal varying levels of technostress among nursing educators, with higher levels observed among older faculty members, frequently associated with limited technical and administrative support. Although the direct impact on job satisfaction was not significant, the anxiety induced by intensive technology use and the perceived necessity for constant professional updating—often driven by FoMO—was shown to affect daily academic life, highlighting the need for effective coping strategies. Understanding technostress within the context of nursing education is essential for addressing the challenges of pedagogical modernization. This review supports the need for future institutional interventions aimed at preventing technostress and fostering a more balanced, reflective, and sustainable relationship with technology in academic settings.

## 1. Introduction

Technological advancement has profoundly transformed higher education, requiring faculty members to continuously adapt to new tools, digital methodologies, and innovative pedagogical demands. In this context, the phenomenon of fear of missing out (FoMO) has gained relevance, often manifesting through a range of negative emotional states [1]. FoMO related to technological evolution emerges as a significant contributor to technostress [2,3,4], manifesting through feelings such as fear of exclusion from ongoing digital innovation in academic settings, anxiety, insecurity, work overload, technophobia, exhaustion, reduced productivity, and job dissatisfaction [5,6,7].

The COVID-19 pandemic and the accelerated digitalization process intensified the phenomenon of technostress, forcing a sudden and widespread adoption of technologies for distance learning. This shift has increased the pressure on educators to maintain their effectiveness in a digital environment, often without sufficient time or resources for sustained and well-supported innovation [5,8,9].

Technostress refers to the stress experienced by individuals as a result of their interaction with digital technologies [10,11]. Initially introduced in the 1980s, the term described the difficulties some individuals faced in adapting to the introduction of computers in the workplaces. However, over the past decades, technostress has evolved into a complex and widespread phenomenon, particularly in the context of rapid digitalization and the increasing integration of technology into all areas of life.

Modern workplaces now rely heavily on digital systems, artificial intelligence, automation, and real-time communication platforms, which demand constant adaptability and cognitive engagement from individuals.

This continuous exposure to technological demands can lead to various psychological, emotional, and behavioral consequences [12]. Technological stress can lead to symptoms such as fatigue, anxiety, poor sleep, decreased job satisfaction or productivity—and can sometimes lead to psychological burnout. These symptoms are often aggravated by factors such as information overload, constant connectivity, lack of digital skills, frequent system updates, and the pressure to perform efficiently in technology-driven environments [13,14,15].

Technostress can be categorized into several dimensions, including techno-overload (the pressure to work faster and handle more information); techno-invasion (the intrusion of technology into personal life); techno-complexity (the need to learn and understand complex systems); techno-insecurity (fear of job loss due to automation); and techno-uncertainty (constant changes in digital tools and platforms) [16,17]. These dimensions reflect the multifactorial nature of technostress and its varied impact on individual well-being and organizational dynamics.

Recognizing technostress as a contemporary occupational health issue is essential for developing strategies to mitigate its effects [12]. Interventions may include promoting digital literacy, implementing organizational policies that regulate digital demands, fostering healthy boundaries between professional and personal life, and creating supportive environments that prioritize employee well-being in the face of ongoing technological change.

In the context of higher education, the digitalization of pedagogical practices has redefined the professional identity and expectations placed upon academic staff. The demand for digital proficiency extends beyond teaching delivery, encompassing virtual student engagement, digital content creation, and the use of learning management systems, often without sufficient time for training or pedagogical reflection [18]. This acceleration in digital expectations can challenge the traditional roles and autonomy of faculty members, leading to increased cognitive load and professional disorientation. As institutions adopt more data-driven and technology-mediated processes, educators may also encounter conflicting demands between administrative efficiency and pedagogical values, further intensifying stress [3]. Institutional culture plays a key role in how academic staff experience technostress. When new technologies are introduced without involving teachers in the decision-making process or considering differences between academic disciplines, educators may see these changes as imposed rather than helpful [19]. This lack of involvement can lead to frustration, emotional fatigue, and disengagement [19,20]. On the other hand, when institutions encourage collaboration, offer regular peer support, and acknowledge different levels of digital skills, teachers are more likely to feel supported and to adapt more effectively to technological change [18].

Health educators are particularly affected by digitalization due to the need to remain constantly updated with rapidly evolving clinical knowledge and technological advancements. In addition to mastering digital tools for effective teaching, they must also integrate new clinical content and innovations to ensure relevant and engaging instruction. This dual demand increases their exposure to technostress and underscores the importance of tailored institutional support. Nurse educators, in particular, must constantly update both their clinical knowledge and pedagogical methods, often balancing practical clinical responsibilities with the rapid pace of technological and educational innovation. This unique intersection makes them especially susceptible to technostress compared to other academic disciplines, reinforcing the need for tailored support and focused research.

Given these influences, a scoping review is necessary to identify the best available evidence on technology stress among nursing faculty in higher education.

## 2. Materials and Methods

This scoping review was conducted following the Joanna Briggs Institute’s (JBI) methodology for conducting scoping reviews [21,22], ensuring a comprehensive and systematic exploration of the scientific literature. The process was reported in accordance with the Preferred Reporting Items for Systematic Reviews and Meta-Analyses extension for Scoping Reviews (PRISMA-ScR) [23], underscoring our commitment to transparency and reproducibility. The review protocol was registered in Open Science Framework [osf.io/2wtmc].

### 2.1. Review Questions

This scoping review aimed to map the existing scientific evidence on technostress among higher education nursing faculty members. The review was guided by the following research questions:

- What factors contribute to the development and experience of technostress in this population?
- What instruments have been used to assess technostress in nursing educators?
- What impact does technostress have on the professional life of nursing educators, and what coping strategies or institutional responses have been reported?

### 2.2. Eligibility Criteria

The eligibility criteria were defined using the PCC mnemonic (Population, Concept, and Context):

- Population: Academic nursing staff with teaching responsibilities in higher education institutions;
- Concept: Technostress;
- Context: Higher education settings (undergraduate, master’s, and doctoral levels).

This review included primary studies with qualitative or quantitative methodologies, written in English, Portuguese, or Spanish, irrespective of publication date. Both published and unpublished literature were considered.

### 2.3. Information Sources and Search Strategy

The search strategy followed three steps, as recommended by the JBI methodology. First, an initial exploratory search was conducted in PROSPERO, OSF, MEDLINE (via PubMed), the Cochrane Database of Systematic Reviews, and the JBI Evidence Synthesis to identify relevant keywords and indexing terms. Then, a comprehensive database search was performed using all identified descriptors and keywords. The following databases were searched: MEDLINE (via PubMed), CINAHL Complete, Scopus, Academic Search Complete, MedicLatina, Psychology and Behavioral Sciences Collection, ERIC, and Teacher Reference Center. Finally, a manual search of reference lists from included studies was carried out to identify additional eligible publications.

The full search was conducted on 21 October 2024. The exact search strategies and applied filters for each database are provided in Table 1.

### 2.4. Study Selection

All retrieved references were imported into Mendeley^®^ for deduplication. Titles and abstracts were screened independently by two reviewers. Full-text articles were obtained for studies that met the inclusion criteria or in cases of uncertainty. Disagreements were resolved through discussion or, when necessary, by consulting a third reviewer. Studies identified through reference lists were screened using the same eligibility criteria.

### 2.5. Data Extraction and Analysis

Data extraction was performed independently by two reviewers using a standardized data charting form aligned with the review objectives and research questions. Extracted data included author(s), year of publication, country, study design, objectives, sample characteristics, instruments used, its impact on academic practice, and any contributing factors or coping strategies. These data were charted using a structured table developed by the research team. Discrepancies were resolved by discussion or a third reviewer.

Data were synthesized narratively and organized thematically to map the scope, focus areas, and gaps in the current literature.

## 3. Results

**Figure 1:**
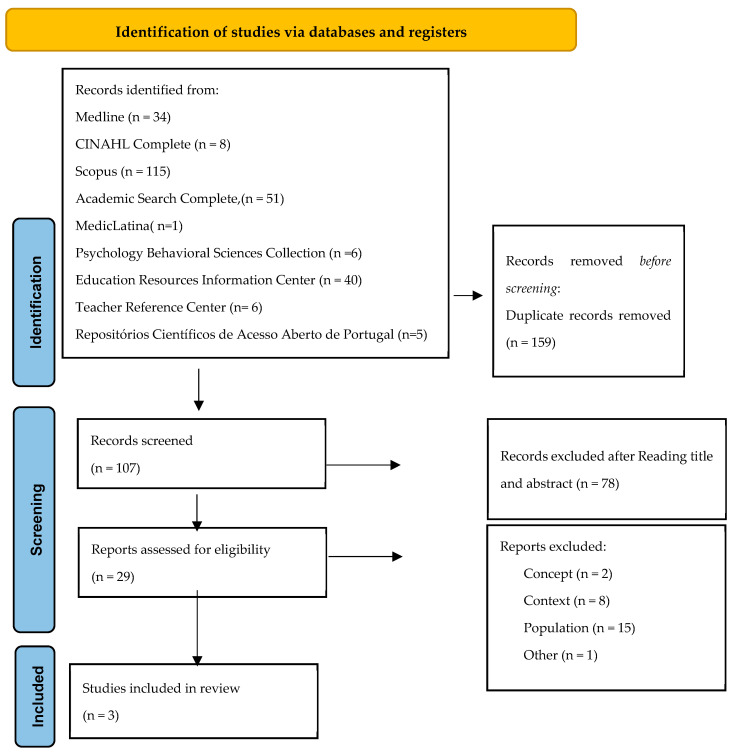
Flow diagram (adapted from [24]).

As presented in Figure 1, the search identified a total of 266 potentially relevant studies. Of these, 159 were excluded as duplicates. From the remaining 107 studies, 78 were excluded after title and abstract screening. Of the 29 full-text articles assessed for eligibility, 26 were excluded for not meeting the inclusion criteria. As a result, three studies were included in this scoping review.

Three studies were included in the review: two conducted in the United States—by Burke (2009) [25] and by Tacy, Northam, and Wieck (2016) [26]— and one in the Philippines by Banaticla and Yango (2023) [27]. All studies adopted a descriptive correlational design. The following sections present a narrative synthesis of the results, organized according to the three guiding research questions. Table 2 presents the detailed characteristics and main findings of the included studies.

### 3.1. Factors Contributing to the Development and Experience of Technostress

All three studies identified excessive workload demands and rapid technology change as the main drivers of technostress among nursing faculty. These factors were primarily linked to institutional, technological, and individual dimensions.

Burke [25] reported that aging faculty experienced moderate-to-high technostress, largely due to the need to learn and prepare digital materials for multiple platforms simultaneously, leading to feelings of overload and frustration. Tacy, Northam, and Wieck [28] found that techno-overload was the strongest negative predictor of technology adoption (β = −0.45, *p* < 0.001), while techno-uncertainty—manifested as anxiety over frequent software updates—and techno-invasion—when work demands intruded on personal time—also contributed significantly to FoMO in online teaching contexts.

Banaticla and Yango [27] extended this by demonstrating that higher levels of perceived technostress were strongly correlated with generalized anxiety (r = 0.62, *p* < 0.001), indicating that inability to manage evolving teaching technologies provokes measurable distress.

Overall, the results suggest that technostress arises from a complex interaction between technological demands and the perceived lack of support or time to meet them.

### 3.2. Instruments Used to Assess Technostress

Each study employed psychometrically sound instruments to quantify technostress and its correlates. The most commonly instrument used was the Nurse Educator Technostress Scale (NETS), applied in both Burke [25] and Tacy et al. [26]. This scale was specifically designed to assess technostress among nurse educators and demonstrated high internal consistency.

In addition to NETS, Tacy et al. [26] employed four other tools:

- The Technology Acceptance Model (TAM) scales, measuring perceived usefulness, perceived ease of use, attitude toward technology, behavioral intention, and system use;
- The Attitudes Toward E-Learning Tool (ATEL);
- The Job in General and Job Descriptive Index scales.

Banaticla and Yango [27] developed a questionnaire divided into three thematic components, technostress, anxiety, and coping strategies, although no standardized or validated instrument was explicitly mentioned.

### 3.3. Impact and Coping Strategies

Technostress has demonstrable negative repercussions for nursing educators’ professional performance and well-being. All three studies describe emotional distress—frustration, anxiety, and diminished motivation—as well as worries about personal efficacy and the ability to meet institutional expectations. In Burke [25], technostress was particularly pronounced among senior faculty lacking administrative support, who reported high levels of frustration and low motivation. Tacy et al. [26] found that, although technostress was only a modest predictor of job satisfaction and retention intention, it significantly undermined perceived ease of use and increased professional strain, leading to reduced teaching engagement. Banaticla and Yango [27] were the only authors to explore coping strategies empirically, showing that educators relied on individual mechanisms—time management, emotional self-regulation, and peer support—to manage stress and sustain functionality in digitally demanding environments. No study, however, evaluated formal institutional interventions to mitigate technostress. These findings highlight a critical gap; the absence of structured, organization-level responses compels reliance on personal coping strategies to confront an escalating professional challenge.

## 4. Discussion

Nursing education in higher education is increasingly defined by technological overload, the complexity of digital tools and anxiety stemming from relentless updates. The studies by Burke (2009), Tacy et al. (2016), and Banaticla and Yango (2023) converge on the experience of frustration, diminished motivation, and concerns about professional performance, demonstrating that technostress transcends mere technical challenges to undermine educators’ mental well-being and pedagogical effectiveness [25,26,27]. These results are consistent with Molino et al. (2020), who identified techno-overload and techno-invasion as key stressors among university faculty, leading to emotional exhaustion and reduced engagement [28], and with Leung and Zhang (2021), who showed that unsupportive organizational climates amplify the negative effects of technostress on academic staff’s health and retention intentions [29].

Burke reported that 68% of nursing educators experienced moderate to high technostress when preparing lessons, especially among senior faculty without adequate administrative support, resulting in frustration and low motivation [25]. Ragu-Nathan et al. (2008) similarly found that poor infrastructure and organizational backing intensify technostress and foster both emotional exhaustion and turnover intentions among knowledge workers [30]. Tacy et al. demonstrated that, although technostress was only a moderate predictor of job satisfaction and intention to stay, it significantly weakened the perceived ease of use of technology (β = −0.32, *p* < 0.01) and heightened professional strain, thereby reducing engagement with online teaching [26]. In parallel, Tarafdar, Tu, and Ragu-Nathan (2007) observed that techno-complexity and techno-uncertainty directly increase role stress and predict work exhaustion across digital professions [31].

Banaticla and Yango highlighted a strong association between technostress and generalized anxiety (r = 0.62, *p* < 0.001), indicating that failure to keep pace with digital innovations can provoke clinical levels of emotional distress [27]. Salanova, Llorens, and Cifre (2013) similarly documented that chronic exposure to information and communication technology demands correlates with higher burnout and anxiety symptoms in educators [32], while O’Doherty et al. (2021) described “digital fatigue” as an emerging construct, marked by cognitive overload and reduced teaching effectiveness during prolonged online instruction [33]. Only Banaticla and Yango empirically evaluated coping strategies, showing that effective time management, emotional self-regulation, and peer support each exerted a significant anxiety-reducing effect (*p* < 0.05) [27]. Zheng et al. (2022) further demonstrated that structured digital literacy workshops coupled with ongoing mentorship can significantly decrease technostress levels among academic staff [34]. In contrast, Burke and Tacy et al. offered untested recommendations—such as instructional design workshops, mentoring schemes, and dedicated helpdesk services—without evaluating their outcomes.

This body of evidence underscores a critical gap: the scarcity of rigorously assessed, institution-level interventions tailored to nursing educators. Reliance on individual coping mechanisms, in the absence of proven organizational support structures, appears insufficient to address the escalating demands of digital teaching environments.

### 4.1. Practical Implications

The evidence indicates that institutions must move beyond ad hoc technical training to embrace comprehensive support programs. Continuous professional development in the usability of digital platforms, grounded in technology acceptance theory, will bolster educators’ sense of control and technological competence.

Drawing on the literature, institutions may implement comprehensive digital mentoring programs, establish peer-support networks, and schedule regular digital well-being workshops. Examples include structured ‘digital detox’ periods, participatory decision-making regarding technology adoption, and ongoing professional development focused on both technological and emotional resilience.

Intergenerational mentoring arrangements, in which digitally adept staff guide more experienced colleagues, can alleviate techno-complexity and cultivate academic social capital. Furthermore, establishing digital detox practices—such as defined notification-free periods and clear switch-off times—will help preserve boundaries between work and personal life, reducing the risk of burnout and maintaining long-term well-being.

### 4.2. Limitations and Future Research

This review is constrained by the small number of available studies and their predominantly cross-sectional designs, which preclude causal inference. Methodological heterogeneity and sample variability further restrict the generalizability of findings. Given that FoMO emerges as a central psychological mechanism driving technostress, future research should prioritize the development and psychometric validation of tools capable of quantifying FoMO specifically among nurse educators, ensuring sensitivity to interventions. Furthermore, institutions should consider integrating awareness and training on FoMO management within digital literacy programs, as addressing this psychological driver may contribute to more effective mitigation of technostress.

Rigorous randomized controlled trials of institutional support initiatives are also needed, evaluating both technological uptake and outcomes related to educator well-being and job satisfaction. Additionally, this review was limited by the geographic concentration of the included studies and by a reliance on published, peer-reviewed sources, which may not capture relevant gray literature or perspectives from underrepresented regions. Future scoping reviews may benefit from a more extensive search of gray literature sources—such as dissertations, conference abstracts, and institutional reports—to identify additional relevant studies and insights that may not be represented in peer-reviewed databases.

Although the protocol for this scoping review was registered with the Open Science Framework (OSF), registration was completed retrospectively, at the time of manuscript submission. This may introduce some risk of reporting bias; however, retrospective registration was undertaken to enhance the transparency and credibility of the review process.

Finally, investigations into contextual factors—such as organizational culture and the level of institutional backing—will clarify how these variables modulate the relationship between technostress and professional performance. In the meantime, institutional leaders and policymakers should consider promoting targeted digital literacy training, participatory technology implementation, and structured peer support to mitigate technostress among nursing educators. Primary studies are needed to strengthen the evidence base and clarify the effectiveness of these strategies in diverse educational settings.

## 5. Conclusions

Technostress emerges as a determining factor for both the quality of instruction and the mental health of nursing educators in higher education. While current evidence endorses integrated approaches combining skills training, social support, and digital work balance strategies, there remains an urgent need for empirical evaluation of these measures. Implementing structured institutional interventions represents the essential next step towards cultivating a more resilient and sustainable academic environment.

## Figures and Tables

**Table 1:** Database search strategy and results.

Database	Query	Record Retrieved
Medline (via PubMed)	((((((((((((((“Higher Education”[Title/Abstract]) OR (“Postsecondary Education”[Title/Abstract])) OR (“Tertiary Education”[Title/Abstract])) OR (“Academic Institutions”[Title/Abstract])) OR (“Graduate Education”[Title/Abstract])) OR (“Undergraduate Education”[Title/Abstract])) OR (Academia[Title/Abstract])) OR (University[Title/Abstract])) OR (Universities[Title/Abstract])) OR (College[Title/Abstract])) OR (Colleges[Title/Abstract])) OR (“Education, Graduate”[Mesh])) OR (“Universities”[Mesh])) OR (“Faculty”[Mesh])) AND (((((((((((((((“Fear of missing out”[Title/Abstract]) OR (FoMO[Title/Abstract])) OR (“Techno-Anxiety”[Title/Abstract])) OR (Techno-Stress[Title/Abstract])) OR (“Technological stress”[Title/Abstract])) OR (Technostress[Title/Abstract])) OR (Information Overload[Title/Abstract])) OR (Infoxication[Title/Abstract])) OR (“Digital Adaptability”[Title/Abstract])) OR (Techno-distress[Title/Abstract])) OR (“Techno-Eustress”[Title/Abstract])) OR (“frustration with technology”[Title/Abstract]))))) AND ((((((((“Education, Professional”[MeSH Terms]) OR (“Education, Professional”[Title/Abstract])) OR (Mentoring[Title/Abstract])) OR (Preceptorship[Title/Abstract])) OR (Educator[Title/Abstract])) OR (Professor[Title/Abstract])) OR (Teacher[Title/Abstract])) OR (Educational Personnel[MeSH Terms])) AND ((medline[Filter]) AND (english[Filter] OR portuguese[Filter] OR spanish[Filter])) AND ((medline[Filter]) AND (english[Filter] OR portuguese[Filter] OR spanish[Filter])) Filters: English, Portuguese, Spanish, MEDLINE	34
CINAHL Complete	(TI Teacher OR AB Teacher OR TI Professor OR AB Professor OR TI Educator OR AB Educator OR TI Preceptorship OR AB Preceptorship OR TI Mentoring OR AB Mentoring OR TI “Education, Professional” OR AB “Education, Professional”) AND ((TI “Fear of missing out” OR AB “Fear of missing out” OR TI FoMO OR AB FoMO OR TI “Technological Anxiety” OR AB “Technological Anxiety” OR TI “Techno-Anxiety” OR AB “Techno-Anxiety” OR TI Techno-Stress OR AB Techno-Stress OR TI “Technological stress” OR AB “Technological stress”) OR (TI Technostress OR AB Technostress OR TI Information Overload OR AB Information Overload OR TI Infoxication OR AB Infoxication OR TI “Digital Adaptability” OR AB “Digital Adaptability” OR TI Techno-distress OR AB Techno-distress OR TI “Techno-Eustress” OR AB “Techno-Eustress”) OR (TI “frustration with technology” OR AB “frustration with technology”)) AND ((TI “Higher Education” OR AB “Higher Education” OR TI “Postsecondary Education” OR AB “Postsecondary Education” OR TI “Tertiary Education” OR AB “Tertiary Education” OR TI “Academic Institutions” OR AB “Academic Institutions” OR TI “Graduate Education” OR AB “Graduate Education” OR TI “Undergraduate Education” OR AB “Undergraduate Education”) OR (TI Academia OR AB Academia OR TI University OR AB University OR TI Universities OR AB Universities OR TI College OR AB College OR TI Colleges OR AB Colleges) OR MH Education, Graduate OR MH (Colleges and Universities) OR MH Faculty) Filters: English, Portuguese, Spanish.	8
Scopus	TITLE-ABS-KEY ((“Higher Education” OR “Postsecondary Education” OR “Tertiary Education” OR “Academic Institutions” OR “Graduate Education” OR “Undergraduate Education” OR academia OR university OR universities OR college OR colleges) AND (“Fear of missing out” OR fomo OR “Techno-Anxiety” OR “Techno-Stress” OR “Technological stress” OR technostress OR “Information Overload” OR infoxication OR “Digital Adaptability” OR “Techno-distress” OR “Techno-Eustress” OR “frustration with technology”) AND (“Education, Professional” OR mentoring OR preceptorship OR educator OR professor OR teacher)) AND NOT (PMID (1*) OR PMID (2*) OR PMID (3*) OR PMID (4*) OR PMID (5*) OR PMID (6*) OR PMID (7*) OR PMID (8*) OR PMID (9*)) AND (LIMIT-TO (LANGUAGE, “English”) OR LIMIT-TO (LANGUAGE, “Spanish”))	115
Academic Search Complete	((TI “Higher Education” OR TI “Postsecondary Education” OR TI “Tertiary Education” OR TI “Academic Institutions” OR TI “Graduate Education” OR TI “Undergraduate Education” OR TI Academia OR TI University OR TI Universities OR TI College OR TI Colleges OR AB “Higher Education” OR AB “Postsecondary Education” OR AB “Tertiary Education” OR AB “Academic Institutions” OR AB “Graduate Education” OR AB “Undergraduate Education” OR AB Academia OR AB University OR AB Universities OR AB College OR AB Colleges) AND (TI “Fear of missing out” OR TI FoMO OR TI “Techno-Anxiety” OR TI “Techno-Stress” OR TI “Technological stress” OR TI Technostress OR TI “Information Overload” OR TI Infoxication OR TI “Digital Adaptability” OR TI “Techno-distress” OR TI “Techno-Eustress” OR TI “frustration with technology” OR AB “Fear of missing out” OR AB FoMO OR AB “Techno-Anxiety” OR AB “Techno-Stress” OR AB “Technological stress” OR AB Technostress OR AB “Information Overload” OR AB Infoxication OR AB “Digital Adaptability” OR AB “Techno-distress” OR AB “Techno-Eustress” OR AB “frustration with technology”) AND (TI “Education, Professional” OR TI Mentoring OR TI Preceptorship OR TI Educator OR TI Professor OR TI Teacher OR AB “Education, Professional” OR AB Mentoring OR AB Preceptorship OR AB Educator OR AB Professor OR AB Teacher)) Filters: English, Portuguese, Spanish.	51
MedicLatina	((TI “Higher Education” OR TI “Postsecondary Education” OR TI “Tertiary Education” OR TI “Academic Institutions” OR TI “Graduate Education” OR TI “Undergraduate Education” OR TI Academia OR TI University OR TI Universities OR TI College OR TI Colleges OR AB “Higher Education” OR AB “Postsecondary Education” OR AB “Tertiary Education” OR AB “Academic Institutions” OR AB “Graduate Education” OR AB “Undergraduate Education” OR AB Academia OR AB University OR AB Universities OR AB College OR AB Colleges) AND (TI “Fear of missing out” OR TI FoMO OR TI “Techno-Anxiety” OR TI “Techno-Stress” OR TI “Technological stress” OR TI Technostress OR TI “Information Overload” OR TI Infoxication OR TI “Digital Adaptability” OR TI “Techno-distress” OR TI “Techno-Eustress” OR TI “frustration with technology” OR AB “Fear of missing out” OR AB FoMO OR AB “Techno-Anxiety” OR AB “Techno-Stress” OR AB “Technological stress” OR AB Technostress OR AB “Information Overload” OR AB Infoxication OR AB “Digital Adaptability” OR AB “Techno-distress” OR AB “Techno-Eustress” OR AB “frustration with technology”) AND (TI “Education, Professional” OR TI Mentoring OR TI Preceptorship OR TI Educator OR TI Professor OR TI Teacher OR AB “Education, Professional” OR AB Mentoring OR AB Preceptorship OR AB Educator OR AB Professor OR AB Teacher)) Filters: English, Portuguese, Spanish.	1
Psychology and Behavioral Sciences Collection	((TI “Higher Education” OR TI “Postsecondary Education” OR TI “Tertiary Education” OR TI “Academic Institutions” OR TI “Graduate Education” OR TI “Undergraduate Education” OR TI Academia OR TI University OR TI Universities OR TI College OR TI Colleges OR AB “Higher Education” OR AB “Postsecondary Education” OR AB “Tertiary Education” OR AB “Academic Institutions” OR AB “Graduate Education” OR AB “Undergraduate Education” OR AB Academia OR AB University OR AB Universities OR AB College OR AB Colleges) AND (TI “Fear of missing out” OR TI FoMO OR TI “Techno-Anxiety” OR TI “Techno-Stress” OR TI “Technological stress” OR TI Technostress OR TI “Information Overload” OR TI Infoxication OR TI “Digital Adaptability” OR TI “Techno-distress” OR TI “Techno-Eustress” OR TI “frustration with technology” OR AB “Fear of missing out” OR AB FoMO OR AB “Techno-Anxiety” OR AB “Techno-Stress” OR AB “Technological stress” OR AB Technostress OR AB “Information Overload” OR AB Infoxication OR AB “Digital Adaptability” OR AB “Techno-distress” OR AB “Techno-Eustress” OR AB “frustration with technology”) AND (TI “Education, Professional” OR TI Mentoring OR TI Preceptorship OR TI Educator OR TI Professor OR TI Teacher OR AB “Education, Professional” OR AB Mentoring OR AB Preceptorship OR AB Educator OR AB Professor OR AB Teacher)) Filters: English, Portuguese, Spanish.	6
Education Resources Information Center	ERIC—searched on 21 October 2024: 40 results ((TI “Higher Education” OR TI “Postsecondary Education” OR TI “Tertiary Education” OR TI “Academic Institutions” OR TI “Graduate Education” OR TI “Undergraduate Education” OR TI Academia OR TI University OR TI Universities OR TI College OR TI Colleges OR AB “Higher Education” OR AB “Postsecondary Education” OR AB “Tertiary Education” OR AB “Academic Institutions” OR AB “Graduate Education” OR AB “Undergraduate Education” OR AB Academia OR AB University OR AB Universities OR AB College OR AB Colleges) AND (TI “Fear of missing out” OR TI FoMO OR TI “Techno-Anxiety” OR TI “Techno-Stress” OR TI “Technological stress” OR TI Technostress OR TI “Information Overload” OR TI Infoxication OR TI “Digital Adaptability” OR TI “Techno-distress” OR TI “Techno-Eustress” OR TI “frustration with technology” OR AB “Fear of missing out” OR AB FoMO OR AB “Techno-Anxiety” OR AB “Techno-Stress” OR AB “Technological stress” OR AB Technostress OR AB “Information Overload” OR AB Infoxication OR AB “Digital Adaptability” OR AB “Techno-distress” OR AB “Techno-Eustress” OR AB “frustration with technology”) AND (TI “Education, Professional” OR TI Mentoring OR TI Preceptorship OR TI Educator OR TI Professor OR TI Teacher OR AB “Education, Professional” OR AB Mentoring OR AB Preceptorship OR AB Educator OR AB Professor OR AB Teacher)) Filters: English, Portuguese, Spanish	40
Teacher Reference Center	((TI “Higher Education” OR TI “Postsecondary Education” OR TI “Tertiary Education” OR TI “Academic Institutions” OR TI “Graduate Education” OR TI “Undergraduate Education” OR TI Academia OR TI University OR TI Universities OR TI College OR TI Colleges OR AB “Higher Education” OR AB “Postsecondary Education” OR AB “Tertiary Education” OR AB “Academic Institutions” OR AB “Graduate Education” OR AB “Undergraduate Education” OR AB Academia OR AB University OR AB Universities OR AB College OR AB Colleges) AND (TI “Fear of missing out” OR TI FoMO OR TI “Techno-Anxiety” OR TI “Techno-Stress” OR TI “Technological stress” OR TI Technostress OR TI “Information Overload” OR TI Infoxication OR TI “Digital Adaptability” OR TI “Techno-distress” OR TI “Techno-Eustress” OR TI “frustration with technology” OR AB “Fear of missing out” OR AB FoMO OR AB “Techno-Anxiety” OR AB “Techno-Stress” OR AB “Technological stress” OR AB Technostress OR AB “Information Overload” OR AB Infoxication OR AB “Digital Adaptability” OR AB “Techno-distress” OR AB “Techno-Eustress” OR AB “frustration with technology”) AND (TI “Education, Professional” OR TI Mentoring OR TI Preceptorship OR TI Educator OR TI Professor OR TI Teacher OR AB “Education, Professional” OR AB Mentoring OR AB Preceptorship OR AB Educator OR AB Professor OR AB Teacher)) Filters: English, Portuguese, Spanish.	6

**Table 2:** Studies included in the scoping review.

Author/Year/Country	Study Type	Proposed Objectives	Instrument Used	Population	Triggering Factors	Impact and Coping Strategies
Burke, Mary S. (2009, USA) [25]	Quantitative (Descriptive-Correlational)	Describe the technological stressors experienced by Louisiana baccalaureate nurse educators	Online questionnaire: Demographic questionnaire and the Nurse Educator Technostress Scale (NETS)	n = 115 nurse educators	Aging faculty experiencing increased stress due to rapid technological changes; lack of administrative support.	Faculty experienced frustration, reduced motivation, and concerns about their ability to meet teaching demands. These emotional and cognitive impacts affected their sense of professional efficacy. No coping strategies or institutional responses were reported
Banaticla & Yango (2023) Laguna, Philippines [27]	Quantitative (Descriptive-Correlational)	Determine the technostress, anxiety, and coping strategies in online teaching and identify the coping strategies that they employ that help them survive the struggles.	Online questionnaire divided into three parts (technostress, anxiety and coping strategies).	n = 144 nurse educators	Technostress was triggered by increased workload, continuous adaptation to digital technologies, and the pressure to integrate new tools into online teaching. These demands compounded existing teaching responsibilities.	Faculty experienced stress and anxiety that affected their well-being and teaching performance. To cope, they reported using personal strategies such as time management, emotional self-regulation, and seeking peer support. No institutional responses were identified.
Tacy, Northam & Wieck (2016), EUA [26]	Quantitative (Descriptive-Correlational)	Examine the effects of technostress, perceived usefulness, ease of use, and attitudes toward technology-on-technology use, job satisfaction, and intent to leave teaching.	Online questionnaires combining five instruments: - demographic information; - the Nurse Educator Technostress Scale (NETS); - Technology Acceptance Model (TAM) scales (perceived usefulness, perceived ease of use, attitude toward use, behavioral intent, and system use); - Attitudes Toward E-Learning tool (ATEL); - Job in General and Job Descriptive Index.	n = 1017 nursing educators	Faculty experienced technostress triggered by aging-related challenges, institutional pressure for rapid digital integration, and high expectations to adopt electronic platforms for teaching.	Technostress was found to be a weak predictor of technology use and job satisfaction, and had no significant impact on intent to stay in the profession. However, it contributed to reduced perceived ease of use and increased professional strain. No coping strategies or institutional responses were reported.

## Data Availability

The datasets used and/or analyzed during the current study are available from the corresponding author on reasonable request.

## Public Involvement Statement

There was no public involvement in any aspect of this research.

## Guidelines and Standards Statement

This manuscript was drafted against the PRISMA 2020 statement guideline for reporting systematic reviews.

## References

[1] Elhai J.D., Yang H., Montag C. (2021). Fear of missing out (Fomo): Overview, theoretical underpinnings, and literature review on relations with severity of negative affectivity and problematic technology use. Braz. J. Psychiatry.

[2] Costin A.D., Ona A.D. (2023). Assessing Technology-Induced Stress Among Students and Teachers. Appl. Med. Inform. Orig. Res..

[3] Vásquez-Pajuelo L., Rodriguez-Barboza J.R., Bartra-Rivero K.R., Andrade-Díaz E.M., Tuesta-Vila J.A., Obando-Peralta E.C., Alarcón-Villalobos  Y.J. (2024). Assessing The Relationship Between Digital Competencies and Technostress in Higher Education. J. Ecohumanism.

[4] Castellanos-Alvarenga L.M., Miranda Rosas L.F., Quiroz Moya M.S., Sanhueza Burgos C.M. (2024). Regulación emocional y tecnoestrés en docentes de educación superior. una revisión sistemática. Rev. Logos Cienc. Tecnol..

[5] Dahabiyeh L., Najjar M.S., Wang G. (2022). Online teaching during COVID-19 crisis: The role of technostress and emotional dissonance on online teaching exhaustion and teaching staff productivity. Int. J. Inf. Learn. Technol..

[6] Khlaif Z.N., Sanmugam M., Joma A.I., Odeh A., Barham K. (2023). Factors Influencing Teacher’s Technostress Experienced in Using Emerging Technology: A Qualitative Study. Technol. Knowl. Learn..

[7] Zhang Q. (2024). Influence of Technical Pressure on Digital Teaching Innovation of Physical Education Teachers Based on Growth-oriented Thinking and TPACK.

[8] Gabbiadini A., Paganin G., Simbula S. (2023). Teaching after the pandemic: The role of technostress and organizational support on intentions to adopt remote teaching technologies. Acta Psychol..

[9] Mushtaque I., Waqas H., Awais-E-Yazdan M. (2022). The effect of technostress on the teachers’ willingness to use online teaching modes and the moderating role of job insecurity during COVID-19 pandemic in Pakistan. Int. J. Educ. Manag..

[10] La Torre G., Esposito A., Sciarra I., Chiappetta M. (2019). Definition, symptoms and risk of techno-stress: A systematic review. International Archives of Occupational and Environmental Health.

[11] Chiappetta M. (2017). The Technostress: Definition, symptoms and risk prevention. Senses Sci..

[12] Rohwer E., Flöther J.C., Harth V., Mache S. (2022). Overcoming the “Dark Side” of Technology—A Scoping Review on Preventing and Coping with Work-Related Technostress. Int. J. Environ. Res. Public Health.

[13] Nimrod G. (2018). Technostress: Measuring a new threat to well-being in later life. Aging Ment. Health.

[14] La Torre G., De Leonardis V., Chiappetta M. (2020). Technostress: How does it affect the productivity and life of an individual? Results of an observational study. Public Health.

[15] Umair A., Conboy K., Whelan E. (2023). Examining technostress and its impact on worker well-being in the digital gig economy. Internet Res..

[16] Lee Y.K. (2021). Impacts of digital technostress and digital technology self-efficacy on fintech usage intention of Chinese gen Z consumers. Sustainability.

[17] Tarafdar M., Tu Q., Ragu-Nathan B.S., Ragu-Nathan T.S. (2007). The impact of technostress on role stress and productivity. J. Manag. Inf. Syst..

[18] Truța C., Maican C.I., Cazan A.M., Lixăndroiu R.C., Dovleac L., Maican M.A. (2023). Always connected @ work. Technostress and well-being with academics. Comput. Hum. Behav..

[19] Li L., Wang X. (2021). Technostress inhibitors and creators and their impacts on university teachers’ work performance in higher education. Cogn. Technol. Work.

[20] Wang X., Li B. (2019). Technostress among teachers in higher education: An investigation from multidimensional person-environment misfit. Front. Psychol..

[21] Peters M.D., Godfrey C., McInerney P., Munn Z., Tricco A.C., Khalil H. (2020). Scoping reviews. JBI Manual for Evidence Synthesis.

[22] Peters M.D., Godfrey C., McInerney P., Munn Z., Tricco A.C., Khalil H. (2024). Scoping reviews. JBI Manual for Evidence Synthesis.

[23] Tricco A.C., Lillie E., Zarin W., O’Brien K.K., Colquhoun H., Levac D., Moher D., Peters M.D., Horsley T., Weeks L. (2018). PRISMA Extension for Scoping Reviews (PRISMA-ScR): Checklist and Explanation. Ann. Intern. Med..

[24] Page M.J., McKenzie J.E., Bossuyt P.M., Boutron I., Hoffmann T.C., Mulrow C.D., Shamseer L., Tetzlaff J.M., Akl E.A., Brennan S.E. (2021). The PRISMA 2020 statement: An updated guideline for reporting systematic reviews. BMJ.

[25] Burke M.S. (2009). The incidence of technological stress among baccalaureate nurse educators using technology during course preparation and delivery. Nurse Educ. Today.

[26] Tacy J., Northam S., Wieck K.L. (2016). Understanding the Effects of Technology Acceptance in Nursing Faculty: A Hierarchical Regression. https://www.proquest.com/openview/71bdbb917c3d996344c7d88e1c24b64d/1?pq-origsite=gscholar&cbl=2034896.

[27] Banaticla M.S., Yango A.R. (2023). Technostress, anxiety, and coping strategies in online teaching among nurse educators. Tech. Soc. Sci. J..

[28] Molino M., Cortese C., Ghislieri C. (2020). The role of technostress in the job demands-resources model. PLoS ONE.

[29] Leung D., Zhang R. (2021). Organisational support as a moderator of technostress: Evidence from higher education. Educ. Technol. Res. Dev..

[30] Ragu-Nathan T.S., Tarafdar M., Ragu-Nathan B.S., Tu Q. (2008). The consequences of technostress for end users in organizations: Conceptual development and empirical validation. Inf. Syst. Res..

[31] Tarafdar M., Tu Q., Ragu-Nathan B.S. (2007). Impact of technostress on role stress and work exhaustion: A multi-group analysis. J. Comput. Inf. Syst..

[32] Salanova M., Llorens S., Cifre E. (2013). The dark side of technologies: Technostress among users of information and communication technologies. Int. J. Psychol..

[33] O’Doherty D., Dromey M., Lougheed S., Hannigan A., Last J., McGrath D. (2021). Building bridges: Online learning and teaching in higher education during COVID-19. J. Technol. Teach. Educ..

[34] Zheng S., Li H., Du J. (2022). Mitigating technostress through digital-literacy training: Evidence from academic staff. Educ. Inf. Technol..

